# Olfactory function changes and the predictive performance of the Chinese Smell Identification Test in patients with mild cognitive impairment and Alzheimer's disease

**DOI:** 10.3389/fnagi.2023.1068708

**Published:** 2023-02-13

**Authors:** Yan Mi, Xiaojuan Ma, Shan Du, Chengxue Du, Xiaobo Li, Huihui Tan, Jie Zhang, Qi Zhang, Wenzhen Shi, Gejuan Zhang, Ye Tian

**Affiliations:** ^1^Department of Neurology, The Affiliated Hospital of Northwest University, Xi'an No.3 Hospital, Xi'an, Shaanxi, China; ^2^Xi'an Key Laboratory of Cardiovascular and Cerebrovascular Diseases, The Affiliated Hospital of Northwest University, Xi'an No.3 Hospital, Xi'an, Shaanxi, China; ^3^Clinical Medical Research Center, The Affiliated Hospital of Northwest University, Xi'an No.3 Hospital, Xi'an, Shaanxi, China

**Keywords:** mild cognitive impairment, Alzheimer's disease, olfactory function, Chinese Smell Identification Test, early detection

## Abstract

**Objectives:**

Olfactory disorder is one of the sensory features that reflects a decline in cognitive function. However, olfactory changes and the discernibility of smell testing in the aging population have yet to be fully elucidated. Therefore, this study aimed to examine the effectiveness of the Chinese Smell Identification Test (CSIT) in distinguishing individuals with cognitive decline from those with normal aging and to determine whether the patients with MCI and AD show changes in their olfactory identification abilities.

**Methods:**

This cross-sectional study included eligible participants aged over 50 years between October 2019 and December 2021. The participants were divided into three groups: individuals with mild cognitive impairment (MCI), individuals with Alzheimer's disease (AD), and cognitively normal controls (NCs). All participants were assessed using neuropsychiatric scales, the Activity of Daily Living scale, and the 16-odor cognitive state test (CSIT) test. The test scores and the severity of olfactory impairment were also recorded for each participant.

**Results:**

In total, 366 eligible participants were recruited, including 188 participants with MCI, 42 patients with AD, and 136 NCs. Patients with MCI achieved a mean CSIT score of 13.06 ± 2.05, while patients with AD achieved a mean score of 11.38 ± 3.25. These scores were significantly lower than those of the NC group (14.6 ± 1.57; *P* < 0.001). An analysis showed that 19.9% of NCs exhibited mild olfactory impairment, while 52.7% of patients with MCI and 69% of patients with AD exhibited mild to severe olfactory impairment. The CSIT score was positively correlated with the MoCA and MMSE scores. The CIST score and the severity of olfactory impairment were identified as robust indicators for MCI and AD, even after adjusting for age, gender, and level of education. Age and educational level were identified as two important confounding factors that influence cognitive function. However, no significant interactive effects were observed between these confounders and CIST scores in determining the risk of MCI. The area under the ROC curve (AUC) generated from the ROC analysis was 0.738 and 0.813 in distinguishing patients with MCI and patients with AD from NCs based on the CIST scores, respectively. The optimal cutoff for distinguishing MCI from NCs was 13, and for distinguishing AD from NCs was 11. The AUC for distinguishing AD from MCI was 0.62.

**Conclusions:**

The olfactory identification function is frequently affected in patients with MCI and patients with AD. CSIT is a beneficial tool for the early screening of cognitive impairment among elderly patients with cognitive or memory issues.

## 1. Introduction

Alzheimer's disease (AD) is the most common subset of dementia, and its incidence has been steadily rising with the aging of the global population. As a clinical transitional phase to AD, mild cognitive impairment (MCI) affects ~3–42% of the adult population and is associated with a conversion rate ranging from ~2–31%, depending on heterogeneity, diagnostic criteria, and sample characteristics (Seelye et al., 2016). Therefore, the early screening and identification of MCI and prodromal AD using reliable and feasible tools are of paramount significance as they may expand the time window for intervention, retard disease progression, and reduce the burden on the caregiver and economy. Although multiple clinical questionnaires and instruments have been established to detect cognitive decline in patients with MCI or AD, these are limited by low sensitivity, time constraints, a lack of interest by participants, and dependence on hearing or language abilities (De Roeck et al., 2019; Chehrehnegar et al., 2020; Arevalo-Rodriguez et al., 2021). It has been well-established that MCI and AD refer to a series of changes involving cognitive functions, daily life, neuropsychiatric abilities, and neuropathological parameters (Petersen et al., 2018). Therefore, the complete delineation of the potential structural and functional changes in multiple areas of MCI may lead to the development of effective detection tools.

Olfactory alternation, especially olfactory identification impairment, is a characteristic of sensory decline in cognitive functions (Jung et al., 2019). It has been demonstrated that olfactory dysfunction appears years before motor symptoms and cognitive decline in several neurodegenerative diseases, including Parkinson's disease (PD) and AD (Marin et al., 2018). This dysfunction is evident in the early stages of subjective cognitive decline (SCD) and becomes increasingly severe as the disease progresses within the AD spectrum (Wang et al., 2021). The neuroanatomical structures responsible for olfactory processing are smaller in patients with AD. Furthermore, this structural reduction can also be detected at the MCI stage (Jobin et al., 2021a). The neuroanatomical structures of the olfactory system mainly encompass olfactory sensory neurons (OSNs) located in the olfactory epithelium (OE) in the nasal cavity (peripheral olfactory system), the olfactory cortices (central olfactory system), and a synaptic adaptor system called the olfactory bulbs (OBs) that interconnect the other two components with the axons of OSNs and mitral cell dendrites (Dibattista et al., 2020). The odorant receptor-bearing OSNs perceive and transduce the chemical signals into electrical signals that trigger neurotransmitter release in the OBs, thus, in turn, sending the projections into the olfactory cortices (Son et al., 2021). In the olfactory cortices, the piriform cortex is responsible for odor perception, and the amygdala/entorhinal cortices send projections to the hippocampus and are involved in olfactory emotion and memory formation (Saiz-Sanchez et al., 2020). An MRI detected reduced olfactory bulb and tract volumes in patients with MCI and AD (Thomann et al., 2009). Olfactory impairment was associated with smaller gray matter volumes of the amygdala, the entorhinal cortex, and the hippocampus in MCI (Kamath et al., 2022). Volumetric MRI also demonstrated atrophy of the olfactory cortex in AD (Al-Otaibi et al., 2020).

Moreover, a histochemical analysis revealed the presence of AD hallmarks β-amyloid and tau protein in the OE and the olfactory cortices, including the anterior olfactory nucleus, the olfactory tubercle, and the piriform cortex of patients with MCI and AD. A correlation was also observed between amyloid-*β* levels in the OB and the amyloid phase and the severity of cognitive impairment in these patients (Arnold et al., 2010; Saiz-Sanchez et al., 2015). Senile plaques, neurofibrillary tangles, and neuropil threads were found in the OBs and OE in the autopsy sample of juvenile AD (Shimizu et al., 2022). Therefore, the abnormal olfactory anatomical structures reflect olfactory dysfunction in patients with cognitive decline. A population-based survey in Sweden or Germany using smell identification tests revealed that the prevalence of olfactory dysfunction was 19.1 and 21.6%, respectively (Bramerson et al., 2004; Vennemann et al., 2008). This percentage increased up to 50% in adults aged between 65 and 80 years in the US (Ottaviano et al., 2016). However, it reached as high as 85–90% in the early stage of AD, indicating that olfactory function deteriorates with aging and cognitive decline (Walker et al., 2021). Makizako et al. (2014) conducted olfactory function examinations and comprehensive cognitive assessments on 220 patients with MCI and found that participants with severe olfactory dysfunction exhibited reduced verbal and visual memory abilities when compared with participants with normal olfactory function, even after adjusting for age, sex, educational level, and other cognitive performance scores. A large-scale cohort study comparing 1,430 participants with a 15-month follow-up period showed that olfactory disorder was related to the occurrence of MCI and the progression from MCI to AD (Roberts et al., 2016). A meta-analysis summarized that olfactory identification was more impaired in patients with AD than MCI, and a simple-to-administer test of olfactory identification might be able to detect AD (Roalf et al., 2017). Wheeler and Murphy (2021) found that the combination of odor identification and odor familiarity tests could improve both sensitivity and specificity in predicting the transition of cognitive states. Furthermore, the assessment of olfactory function may serve as a promising non-invasive and low-cost screening tool to supplement MoCA scores for the early detection of cognitive decline (Quarmley et al., 2017).

Several smell identification tests have been developed to measure olfactory function, including the most frequently applied University of Pennsylvania Smell Identification Test (UPSIT), the Connecticut Chemosensory Clinical Research Center (CCCRC) olfactory test, the Sniffin' Sticks olfactory test, and the recently developed Brief Smell Identification Test (B-SIT), which is based on the UPSIT (Wu et al., 2019). However, these olfactory identification tests were based on the preferred odors of the western population. Their performance in distinguishing olfactory decline may vary with the local culture and the patients' familiarity with specific odors (such as clove, rosin, raspberries, and Japanese cypress). To address this issue, Zhou et al. designed and developed the Chinese Smell Identification Test (CSIT; Feng et al., 2019), which contains 16 or 40 local odor items that are familiar and identifiable to most Chinese people. The CSIT achieved good performance for assessing olfactory function in the general Chinese population with test-retest reliability of up to 0.92 and was validated against the Sniffin' Sticks test and the UPSIT. The CSIT has been used in assessing the olfactory identification function of patients with major depressive disorder and the inherited metabolic disease Alström syndrome (Wang et al., 2020; Zhang et al., 2022). A previous study demonstrated that a CSIT examination containing 40 odors could be potentially applied to improve the detection sensitivity of Parkinson's disease using an optimal threshold value of 22.5, yielding a sensitivity and a specificity of 71.1 and 89.3%, respectively (Xing et al., 2021).

Moreover, the 40-odor CSIT was also applied to Chinese participants with cognitive decline and showed good performance in differentiating patients with MCI and patients with AD from normal aging adults based on a relatively small sample; the CSIT score was strongly correlated with volumes of the left precentral gyrus, the inferior frontal gyrus, and the amygdala measured using imaging techniques (Wu et al., 2019). However, the performance of the simplified 16-odor CSIT in screening olfactory dysfunction and in distinguishing patients with MCI and patients with AD from cognitively normal individuals has yet to be determined. In the present study, we evaluated olfactory identification characteristics and their association with cognitive function in patients with AD and patients with MCI using the simplified 16-odor CSIT. We also investigated whether these characteristics could distinguish individuals with normal-aging from patients with MCI and patients with AD in a Chinese population.

## 2. Methods

### 2.1. Participates

Between December 2020 and February 2022, a total of 366 eligible participants aged over 50 years were consecutively enrolled in this cross-sectional study in the Department of Neurology and Memory Clinics of the Xi'an No.3 Hospital. The inclusion criteria were as follows: (1) participants between the age of 50 and 85 years; (2) those with no visual or hearing impairments; and (3) those capable of completing neuropsychiatric tests and who volunteered for the olfactory function test and provided signed and informed consent. The exclusion criteria were as follows: (1) secondary dementia due to another known etiology; (2) severe concomitant intracranial diseases such as encephalitis, meningitis, acute cerebral stroke, seizure, brain tumors, and other diseases; (3) vascular or mixed MCI as assessed by the Hachinski Ischemia Scale (HIS) score of ≥ 4; (4) acute respiratory tract infections or olfactory diseases such as rhinitis and sinusitis within 2 weeks; (5) long-term exposure to pesticides, metal dust, and other volatile substances; and (6) impairment or loss of olfactory function due to other known etiologies, such as head trauma, rhinosinusitis, post-infectious sequelae from COVID-19, medication side effects, and other rare conditions. Demographic data for the eligible participants were collected, including age, gender, educational levels, smoking, alcohol consumption, concurrent diseases, and memory complaints. The participants were asked whether they experienced a subjective memory decline for 3 months or not and whether they were worried about this decline. Meanwhile, they were asked the question “Did your parents ever have Alzheimer's disease/dementia?” to document their family history of AD/dementia. In comparison to the age- and sex-matched population, participants with reduced sleep duration reported subjective sleep disorder, difficulty falling asleep, frequent awakenings, early morning awakenings, and excessive daytime sleepiness. The enrolled participants were categorized into the NC, MCI, and AD groups based on the corresponding diagnostic criteria after carefully considering clinical characteristics, medical history, daily life ability, and neuropsychiatric testing performance.

MCI was diagnosed in accordance with the Petersen criteria, as demonstrated by Petersen et al. (1999) and Marcos et al. (2016): (1) subjective complaints of memory decline or decline of other cognitive functions on self or informant reporting or medical assessment; (2) at least one cognitive domain impairment on neuropsychiatric testing (referring to MoCA scores with a cutoff value of 26) standardized by the patient's age and educational background; (3) intact daily functioning according to the Activity of Daily Living (ADL) scale and being able to retain independence in daily living; and (4) not meeting the criteria for a diagnosis of dementia with a clinical dementia rating (CDR) score of <0.5. AD is assessed according to the criteria published by the National Institute of Neurological and Communicative Disorders and Stroke and the Alzheimer's Disease and Related Disorders Association (NINCDS/ADRDA), including (1) meeting the criteria of possible or probable AD; (2) a mini-mental status examination (MMSE) score of < 24; and (3) a CDR score ranging from 0.5 to 2. Eventually, 188 patients with MCI, 42 patients with AD, and 136 cognitively normal controls (NCs) were diagnosed by trained neurological clinicians and recruited for this study. This study was approved by the ethics committee of Xi'an No.3 Hospital, and informed consent was obtained from all participants.

### 2.2. Neuropsychiatric examination

Cognitive function was assessed using the MMSE, the Montreal Cognitive Assessment (MoCA), and the CDR scales, and daily functioning was measured using the Activity of Daily Living scale. MMSE is one of the most widely used questionnaires used in the aging population, with high acceptability by professionals and researchers as a diagnostic instrument. This scale features 30 items relating to the assessment of orientation, recall, attention, calculation, language, and visuospatial abilities of the participants. A score on the MMSE scale of <24 was used to identify patients with dementia. The MoCA scale covers extensive and comprehensive cognitive domains and elevates the scores of the visuospatial and executive domains. The total score on this scale is 30 points, and the higher the score, the better the cognitive ability. When the number of years of education was ≤12, one more point was added to the original total score, and the final score of <26 indicated cognitive impairment and of 21 indicated dementia (Dautzenberg et al., 2022). The CDR was first established by Charles P. Ughes and his colleagues in 1982 to grade the severity of dementia (Morris, 1993). The participants and their family members were interviewed separately. The test results were divided into five grades (0, 0.5, 1, 2, and 3), thus representing normal cognition, mild cognitive impairment, mild dementia, moderate dementia, and severe dementia, respectively. Higher scores indicate greater cognitive impairment. The ADL is a scale developed by Barthel in the 1960's to assess an individual's daily life ability to assist in the diagnosis of MCI or AD (Mahoney and Barthel, 1965). The total score on this scale is 100; a higher score indicates a more independent status for the individual.

### 2.3. Chinese Smell Identification Test

The Chinese Smell Identification Test (CSIT) containing 40 or 16 odors was developed by Zhou's team in 2019 (Institute of Psychology, Chinese Academy of Sciences). The detailed odor items were reported and the reliability of CSIT was validated against UPSIT and Sniffin' Sticks test in China (Feng et al., 2019). A 16-odor CSIT was used in the present study to evaluate olfactory identification performance. This instrument includes 16 consecutively numbered smelling sticks, which evaporate 16 different odors that are familiar to Chinese people and easy to distinguish. The test was performed on the instrument in a quiet environment with clean air. All participants were disallowed from smoking and were asked to fast for 15 min before the test in a comfortable state. The opened stick was placed ~1 cm in front of the participant's nose and shaken 5–7 times to smell for 3 s; then, the stick was closed. This procedure was repeated until the subject confirmed a clear smell. The interval between two odor presentations was ~30 s. For each odor, the subject chose an answer from the options presenting four similar odors. The whole test took ~5–10 min. The number of correctly identified odors was recorded as the CSIT score. The olfactory function of a subject was stratified into normal impairment, mild impairment, moderate impairment, severe impairment, and olfactory loss based on a report that was automatically generated by the dedicated software installed on the CSIT instrument. The severity of olfactory dysfunction was calculated based on an age- and gender-adjusted regression algorithm developed in a large normal cohort across China investigated by the constructor. Normal olfactory function corresponds to 14–16 points in the CSIT score: mild impairment (11–13), moderate impairment (9–11), severe impairment (7–9), and olfactory loss with a CSIT of ≤ 6. In cases when the score was at the cutoff or was overlapping, the instrument was able to judge according to the virtue of the developed algorithm. The smelling sticks were used before the expiration date and replaced promptly to avoid the unreliability caused by odor volatilization.

### 2.4. Statistical analysis

All data were processed using R software version 4.1.2 (Vienna, Austria) and SPSS version 26.0 (IBM Corp., Chicago, Illinois, USA). Numerical data were expressed as frequency and percentage (%). The difference between the three groups was compared using the chi-squared test or Fisher's exact test, with multiple comparisons between every two groups adjusted using Bonferroni's test. Quantitative data were presented as means ± standard deviation (SD) or medians with quartiles. Statistical differences among multiple groups were compared using the one-way analysis of variance (ANOVA), followed by Bonferroni's *post-hoc* test or the non-parametric Kruskal–Wallis test with Dunn's *post-hoc* test. Spearman's correlation analysis was used to analyze the correlation between the CSIT and MMSE or MoCA scores. Multivariate logistic regression analysis was conducted to investigate the association between the CSIT score, the severity of olfactory function, and cognitive function. Three logistic regression models were fit using stepwise adjustment of confounders identified through univariate analysis or other potential covariates. Restricted cubic spline (RCS) curves were plotted to detect the possible non-linear dependency between the risk of MCI or AD and the CSIT score using three knots at specified locations according to the percentiles of the distribution of CSTI scores. Receiver operator characteristic curves (ROCs) were generated. The area under the curve (AUC) was calculated by taking the clinical diagnosis of MCI or AD as the outcome index and the CSIT score as a predictor. The maximum Youden's index (sensitivity+specificity-1; Youden, 1950; O'Caoimh et al., 2017) was selected as the optimal cutoff point, and a *P*-value of < 0.05 was considered statistically significant. Power analysis was performed using StataSE12.0 software (Stata Corporation, TX, USA) based on a two-sample *t*-test of the means and SD of CIST scores and the sample size in the AD and MCI groups, with regard to the smaller AD sample size than the other two groups. The significance level was set at 0.05 (two-sided). The estimated power of this study was 0.894. When we input the CIST score and sample size in the NC and MCI groups, the estimated power reached 0.982.

## 3. Results

### 3.1. Clinical characteristics of the participants

A total of 366 participants completed all tests and were eligible for this cross-sectional study: 188 patients with MCI, 42 patients with AD, and 136 cognitively normal controls. The demographic characteristics and cognitive scores of the participants are compared in Table 1. There was a significant difference in age across the three groups (*F* statistics = 11.839, *P* = 0.003), with a higher mean age in patients with MCI and patients with AD when compared with the NCs; the age was even higher in patients with AD when compared with patients with MCI. A significant difference was also detected for educational levels among the three groups (Fisher's statistic = 36.936, *P* < 0.001); the proportions of patients with <9 educational years were 28, 49.5, and 71.4% in the NC, MCI, and AD groups, respectively, thus indicating that age and educational levels were two potential confounders affecting the cognitive performance of the population aged over 50 years. No significant differences were detected between the three groups with respect to other sociological features such as marital and living status, physical exercise frequency, smoking, and alcohol consumption status. The three groups of participants showed a comparable incidence of concurrent diseases, including hypertension, diabetes, coronary artery disease, and hyperlipidemia. Approximately half of the enrolled participants reported sleep difficulties, and ~90% reported subjective memory decline; there were no significant differences between the groups in this respect. As expected, the mean MMSE and MoCA scores gradually decreased as the severity of cognitive impairment increased. The participants in the AD and MCI groups had significantly lower scores than those in the NC group (both *P* < 0.001).

**Table 1:** Demographic characteristics of the participants.

**Characteristics**	**Non-MCI**	**MCI**	**AD**	**Statistics**	**P-value**
	**(*****n*** = **136)**	**(*****n*** = **188)**	**(*****n*** = **42)**		
Age (years)	60.43 ± 7.61	62.45 ± 7.15	64.69 ± 9.17	11.839	0.003
Sex				2.379	0.307
Women	55 (40.4%)	88 (46.8%)	15 (35.7%)		
Men	81 (59.6)	100 (53.2%)	27 (64.3%)		
Education				36.935	<0.001
≤6 years	8 (5.9%)	28 (14.9%)	14 (33.3%)		
6–9 years	30 (22.1%)	65 (34.6%)	16 (38.1%)		
9–12 years	53 (39.0%)	59 (31.4%)	9 (21.4%)		
>12 years	45 (33.1%)	36 (19.1%)	3 (7.1%)		
Marital status				5.045	0.220
Married	134 (98.5%)	179 (95.2%)	39 (92.9%)		
Divorced	0 (0%)	2 (1.1%)	0 (0%)		
Widowed	2 (1.5%)	7 (3.7%)	3 (7.1%)		
Living status				1.308	0.517
Living with family members	129 (94.9%)	177 (94.1%)	38 (90.5%)		
Living alone	7 (5.1%)	11 (5.9%)	4 (9.5%)		
Exercise				8.228	0.084
Never	8 (5.9%)	15 (8.0%)	8 (19.0%)		
<3 times/week	53 (39.0%)	66 (35.1%)	11 (26.2%)		
>3 times/week	75 (55.1%)	107 (56.9%)	23 (54.8%)		
Smoking				4.156	0.656
None	107 (78.9%)	132 (70.2%)	30 (71.4%)		
<10 cigarettes/day	4 (2.9%)	9 (4.8%)	2 (8.2%)		
10–20 cigarettes/day	19 (14.0%)	35 (18.6%)	6 (14.3%)		
>20 cigarettes/day	6 (4.4%)	12 (6.4%)	4 (9.5%)		
Alcohol consumption				7.288	0.100
None	121 (89.0%)	155 (82.4%)	39 (92.4%)		
Less	13 (9.6%)	26 (13.8%)	1 (2.4%)		
More	2 (1.5%)	7 (3.7%)	2 (4.8%)		
Hypertension	59 (43.4%)	70 (37.2%)	18 (42.9%)	1.384	0.513
Diabetes	11 (8.1%)	23 (12.2%)	7 (16.7%)	2.788	0.257
Hyperlipidemia	36 (26.5%)	51 (27.1%)	13 (31.0%)	0.332	0.852
Coronary arterial diseases	19 (14.0%)	27 (14.4%)	6 (14.3%)	0.010	1.000
Subjective sleep disorder	66 (48.5%)	94 (50.0%)	19 (45.2%)	0.324	0.840
Family history of AD/dementia	20 (14.7%)	21 (11.2%)	3 (7.1%)	2.001	0.401
Self-reported memory decline	120 (88.2%)	175 (93.1%)	39 (92.9%)	2.341	0.334
Worried about memory decline	38 (27.9%)	66 (35.1%)	18 (42.9%)	3.760	0.151
MMSE	29.02 ± 1.04	27.37 ± 1.66	19.60 ± 4.37	177.268	< 0.001
MoCA	27.10 ± 1.31	21.9 ± 2.60	15.1 ± 4.49	279.808	< 0.001

### 3.2. Olfactory identification test performance for MCI and patients with AD

**Figure 1:**
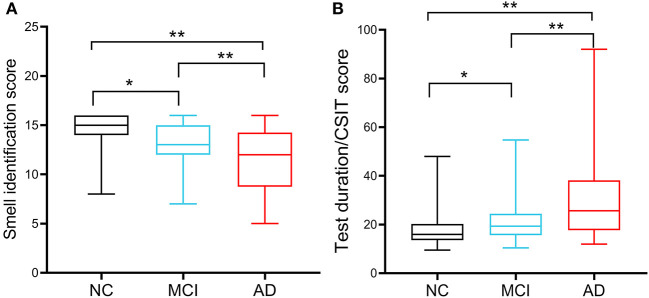
Chinese smell identification score and accurate identification efficiency of the participants. **(A)** Comparison of Chinese smell identification scores in cognitive normal controls (NCs; *n* = 188), patients with MCI (*n* = 136), and patients with AD (*n* = 42). **(B)** Accurate identification efficiency was calculated by the ratio of the used testing time to the achieved scores. *P < 0.05, **P < 0.01.

All participants completed the 16-odor CSIT presented in a multiple-answer format. The number of participants who identified the correct odor in each group is listed and compared in Table 2. The analysis revealed that the correct identification rate in cognitively normal participants exceeded 90% for 12 odors (ranging from 91.2 to 100%), 80–90% for three items, and 67.6% for identifying the longan odor. Longan has a succulent and white aril with a brown seed and is the fruit of *Dimocarpus longan* Lour, a subtropical and tropical evergreen tree belonging to the Sapindaceae family that is widely distributed in China, Southeast Asia, and South Asia (Yue et al., 2022). There was a significant difference in the accuracy rate among the three groups with regards to identifying 13 odors (all *P* < 0.05), except for the odors of orange, almond, and fried fish. When comparing the NC and MCI groups, we observed a significant difference in the identification rate for eight odors (all *P* < 0.05), especially for chocolate, coffee, apple, and lemon (all *P* ≤ 0.001). Compared with the MCI group, patients with AD exhibited a reduced ability to distinguish the odors of garlic, floral water, sesame oil, and longan (all *P* < 0.05). Compared with NC, patients with AD had poor performance in distinguishing 11 odors. Patients with MCI achieved a mean CSIT examination score of 13.06 ± 2.053, while those in the AD group achieved a mean score of 11.38 ± 3.253; these scores were significantly lower than the NC group (14.6 ± 1.57; *P* < 0.001; Figure 1A). The mean times to complete the test were 239.5, 250.50, and 248.50 s for patients in the NC, MCI, and AD groups, respectively. Thus, patients with MCI and AD had a significantly higher mean time-to-score ratio when compared with those in the NC group (19.34 and 25.74 vs. 16.0; Figure 1B). Moreover, olfactory function levels in each individual were automatically assessed using the CSIT instrument and categorized into five subgroups. The analysis demonstrated that 19.9% of participants in the NC had olfactory impairment (mainly mild impairment), while 52.7% of patients with MCI and 69% of patients with AD suffered from olfactory impairment (Fisher's statistic = 65.939, *P* < 0.001; Table 3).

**Table 2:** Comparisons of accuracy in odor identification in each item of CSIT in the three groups.

**Odor items**	**NC (*n* = 136)**	**MCI (*n* = 188)**	**AD (*n* = 42)**	**All cases**		**NC vs. MCI**		**MCI vs. AD**		**NC vs. AD**	
				χ^2^	***P***	χ^2^	*P* ^*^	χ^2^	*P* ^*^	χ^2^	*P* ^*^
Orange	135 (99.3%)	181 (96.3)	40 (95.2%)		0.131		0.217		0.670		0.217
Almond	125 (91.9%)	165 (87.8%)	38 (90.5%)		0.503		0.793		0.793		0.793
Garlic	136 (100%)	188 (100%)	40 (95.2%)		0.013		1.000		0.033		0.055
Chocolate	127 (93.4%)	145 (77.1%)	28 (66.7%)	21.621	<0.001	15.475	<0.001	2.015	0.169	20.355	<0.001
Coffee	132 (97.1%)	153 (81.4%)	30 (71.4%)	24.645	<0.001	18.314	<0.001	2.092	0.202		<0.001
Floral water	131 (96.3%)	183 (97.3%)	32 (76.2%)	31.067	<0.001		0.747	25.190	<0.001	16.856	<0.001
Sesame oil	130 (95.6%)	168 (89.4%)	31 (73.8%)	16.868	<0.001	4.145	0.061	7.120	0.018	17.620	<0.001
Fried fish	120 (88.2%)	161 (85.6%)	31 (73.8%)	5.357	0.069	0.462	0.607	3.483	0.153	5.190	0.126
Banana	127 (93.4%)	157 (83.5%)	32 (76.2%)	10.663	0.005	7.106	0.014	1.256	0.269	9.947	0.011
Rose	119 (87.5%)	136 (72.3%)	27 (64.3%)	14.626	0.001	10.820	0.002	1.079	0.395	11.728	0.002
Anise	125 (91.9%)	170 (90.4%)	33 (78.6%)	6.409	0.041	0.214	0.697	4.656	0.088	5.726	0.074
Apple	110 (80.9%)	93 (49.5%)	20 (47.6%)	36.244	<0.001	33.282	<0.001	0.047	0.963	18.028	<0.001
Longan	92 (67.6%)	102 (54.3%)	15 (35.7%)	14.639	0.001	5.891	0.031	4.722	0.045	13.647	0.001
Lemon	126 (92.6%)	132 (70.2%)	27 (64.3%)	28.126	<0.001	24.485	<0.001	0.565	0.464	21.381	<0.001
Pineapple	127 (93.4%)	160 (85.1%)	33 (78.6%)	8.308	0.016	5.343	0.049	1.086	0.418	7.744	0.038
Soy sauce	124 (91.2%)	161 (85.6%)	31 (73.8%)	8.367	0.015	2.286	0.181	3.483	0.153	8.602	0.023

* Adjusted with Bonferroni's test.

**Table 3:** Functional impairment of olfactory identification ability in the three groups of participants.

**Severity**	**NC**	**MCI**	**AD**
Normal *n* (%)	109 (80.1%)	89 (47.3%)	13 (31.0%)
Mild *n* (%)	22 (16.2%)	65 (34.6%)	12 (28.6%)
Moderate *n* (%)	3 (2.2%)	24 (12.8%)	6 (14.3%)
Severe or loss *n* (%)	2 (1.5%)	10 (5.3%)	11 (26.2%)

### 3.3. Correlations between the CSIT score and cognitive functions in MCI and patients with AD

**Figure 2:**
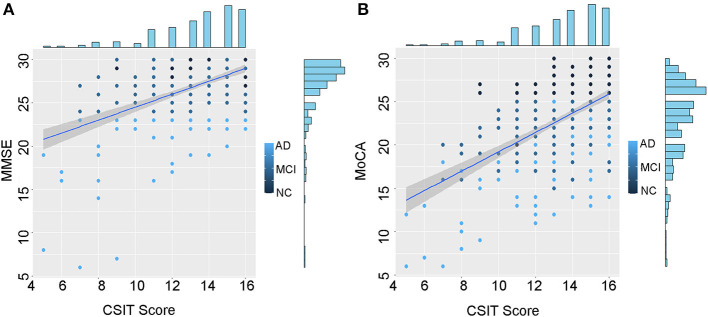
Scatter plots of the CSIT score against MMSE or MoCA in all the included cases. **(A)** Correlation between the CSIT score and the MMSE score and **(B)** correlation between the CSIT score and the MoCA score.

Based on the observed differences in olfactory functions across the three groups, we evaluated the correlation between the CSIT and cognitive scores. Scatter plots were used to illustrate the CSIT scores against MMSE or MoCA scores for everyone in the whole population (Figures 2A, B). Spearman's analysis showed that the correlation coefficients between the CSIT score and the MMSE or MoCA were 0.50 [95% confidence interval (CI): 0.42 – 0.57, *P* < 0.001] and 0.56 (95% CI: 0.49 – 0.63, *P* < 0.001), respectively. For participants in the NC group, CSIT significantly correlated with MoCA (*r* = 0.291, *P* < 0.001) rather than MMSE (*r* = 0.130, *P* = 0.092). In the MCI group, the correlation coefficients between CSIT and MOCA/MMSE scores were 0.459 (*P* < 0.001) and 0.346 (*P* < 0.001). In the AD group, the coefficients between CSIT and MOCA/MMSE were 0.468 (*P* < 0.001) and 0.431 (*P* < 0.001).

### 3.4. Association between olfactory identification ability and cognitive function in MCI and AD

**Figure 3:**
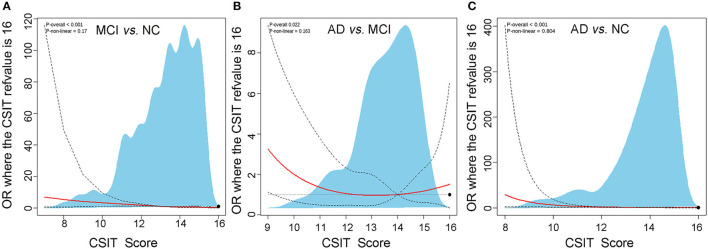
The restricted cubic spline analysis of the potential non-linear association between CSTI score and risk of MCI and AD. **(A)** Adjusted logistic regression algorithm-based restricted cubic spline curve showing the association between CSIT and OR of MCI using three knots at specified locations according to the percentiles of the distribution of the CSTI score. **(B)** Restricted cubic spline plot of the association between CSIT and OR of AD differentiating from MCI using three knots. **(C)** Restricted cubic spline plot of the association between CSIT and OR of AD differentiating from NC using three knots.

Subsequently, we analyzed the association between olfactory identification ability and cognitive function using logistic regression by separately modeling two independent variables, the CSIT score and the olfactory impairment level, in a crucial model and two adjusted models with cognitive status (NC, MCI, or AD) as dependent variables. In the crucial logistic model, only the CSIT score or olfactory impairment level was fitted, and the odds ratio (OR) with a 95% CI and a *P*-value were shown. In the adjusted models, age, gender, educational levels, and concurrent diseases were further adjusted as confounders in stepwise logistic regression. As shown in Table 4, the CSIT score was significantly associated with MCI (OR: 0.59; 95% CI, 0.50–0.69; *P* < 0.001); furthermore, the OR increased to 0.81 (95% CI: 0.69–0.95, *P* = 0.008) after adjusting for the confounders of age, gender, and educational levels but remained unchanged when we adjusted for concurrent diseases in addition to the covariates in Model 2, thus indicating that the higher the CSIT score, the lower the probability of MCI. When modeling olfactory impairment level as an ordinal categorical variable, each stepwise elevation of olfactory rank increased the risk of MCI by 1.83-fold (*P* = 0.008); this significant association remained robust after adjusting for age, gender, educational levels, and other covariates. Similarly, a significant association between olfactory performance and cognitive function was also observed when comparing MCI with AD and AD with NC, regardless of whether we modeled the olfactory function as a continuous or rank predictor. This association remained significant after subtracting the effects of age, gender, and educational levels (Model 2) in addition to other covariates (Model 3). A restricted spline curve (RSC) was generated to illustrate the potential non-linear association between the CSIT score and the OR of MCI when compared with NC after adjusting for age and educational levels (Figure 3A). The curve illustrated a linear relationship between these variables (*P*_non − linear_ = 0.17) and the risk of MCI increased as the CSIT score decreased, especially when the CSIT score was < 10 points. In the adjusted RSC, when differentiating AD from MCI, a decreasing trend line showed that a CSIT score of < 13 points led to an increase in the risk of AD (Figure 3B). The RSC curve analysis also revealed the reduced risk of AD with an increasing CSIT score when distinguishing AD from NC (Figure 3C).

**Table 4:** Logistic analysis of the association between the CSIT score and olfactory impairment severity with the risk of MCI and AD.

	**MCI vs. NC**		**MCI vs. AD**		**AD vs. NC**	
	**CSIT score**	**Olfactory impairment severity**	**CSIT score**	**Olfactory impairment severity**	**CSIT score**	**Olfactory impairment severity**
**Model 1** ^*^						
OR [95%CI]; *P*	0.59 [0.50, 0.69], *P* < 0.001	2.83 [1.92, 4.17], *P* < 0.001	0.76 [0.67, 0.87]; *P* < 0.001	1.84 [1.35, 2.52], *P* < 0.001	0.55 [0.44, 0.67]; *P* < 0.001	3.69 [2.31, 5.90], *P* < 0.001
**Model 2** ^*^						
OR [95%CI]; *P*	0.62 [0.53, 0.73]; *P* < 0.001	2.35 [1.58, 3.50]; *P* < 0.001	0.81 [0.69, 0.95]; *P* = 0.008	1.55 [1.09, 2.21]; *P* = 0.015	0.59 [0.47, 0.74]; *P* < 0.001	2.81 [1.65, 4.77]; *P* < 0.001
**Model 3** ^*^						
OR [95%CI]; *P*	0.61 [0.52, 0.72]; *P* < 0.001	2.38 [1.59, 3.57]; *P* < 0.001	0.80 [0.68, 0.93]; *P* = 0.005	1.61 [1.12, 2.30]; *P* = 0.010	0.58 [0.45, 0.73]; *P* < 0.001	2.88 [1.67, 4.97]; *P* < 0.001

* Model 1: non-adjusted model.

* Model 2: adjusted for age, gender, and education levels.

* Model 3: further adjusted for hypertension, hyperlipidemia, diabetes, CAD, and sleep disorder based on Model 2.

### 3.5. Subgroup analysis and the interaction effect of education and age on the CSIT score in predicting MCI or AD

Later, we performed a subgroup analysis to further investigate the effects of age and education on the relationships between CSIT and the risk of MCI or AD. The participants were stratified into three intervals of age and four subgroups based on their educational background; a cross-table listing the CSIT scores of patients in the NC, MCI, and AD groups is given in Table 5. We found that there were significant differences among the NC, MCI, and AD groups in each layer when stratified by age and educational background. Furthermore, when performing comparisons among the age subgroups, the patients achieved comparable CSIT scores in the NC or MCI group. However, a comparison of CSIT scores across educational levels for patients in the NC, MCI, or AD groups revealed a significant difference; patients with a higher education level tended to achieve higher scores, especially those in the NC and MCI groups. An interaction effect between educational level and CSIT was further investigated using a logistic regression model by adding the constructed variable *education level* × *CSIT* in the regression formula. The coefficients of the constructed variable were 0.99 (95% CI: 0.96–1.01, *P* = 0.305) when classifying MCI from NC and 1.02 (95% CI: 1.00–1.04, *P* = 0.039) while classifying MCI from AD, thus indicating that there was no significant effect of educational level on CSIT when predicting MCI. However, the educational level had an interactive effect on CSIT in predicting AD.

**Table 5:** Subgroup comparison of the CSIT score stratified by age and educational levels.

**Subgroups**	**NC**	**MCI**	**AD**	**Statistics**	***P***
**Age**					
50–60 years	14.63 ± 1.54	13.46 ± 1.77	13.50 ± 1.79	10.040	<0.001
60–70 years	14.69 ± 1.33	12.80 ± 2.21	11.32 ± 2.96	21.935	<0.001
>70 years	14.58 ± 1.62	12.69 ± 2.20	8.22 ± 3.19	20.501	<0.001
Statistics	0.001	4.196	12.486		
*P*	1.000	0.123	0.002		
**Education**					
≤6 years (*n* = 50)	14.50 ± 1.41	12.36 ± 2.28	9.86 ± 3.76	10.905	0.004
6–9 years (*n* = 111)	14.03 ± 1.96	12.69 ± 2.09	11.00 ± 2.78	16.247	<0.001
9–12 years (*n* = 121)	14.68 ± 0.99	13.37 ± 1.84	14.22 ± 1.56	14.866	0.001
>12 years (*n* = 84)	15.04 ± 1.46	13.75 ± 1.90	12.00 ± 1.73	19.529	<0.001
Statistics	8.678	11.091	10.407		
*P*	0.034	0.011	0.015		

### 3.6. Diagnostic value of the CSIT for predicting MCI and AD

**Figure 4:**
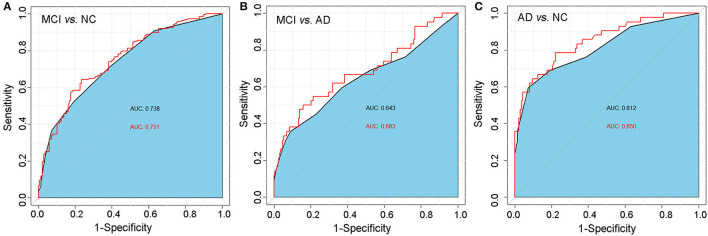
ROC analysis of the predictive value of the CSIT score in distinguishing MCI or AD from normal-aging adults. **(A)** MCI vs. NC; **(B)** AD vs. NC; **(C)** MCI vs. AD.

Finally, we used the ROC analysis to assess the diagnostic value of the CSIT score for classifying MCI or AD. The analysis showed that the AUC for the CSIT score when classifying MCI from NC was 0.738, with a sensitivity of 0.521 and a specificity of 0.801, with an optimal cutoff score of 13; the AUC increased to 0.751 when combined with age and educational level (Figure 4A). A CSIT score at a cutoff point of 10 could predict AD from MCI with an AUC of 0.643; this increased to 0.683 when modeled in combination with age and educational level (Figure 4B). The AUC of the ROC curve reached 0.812 for the CSIT score in classifying AD from NC; it increased to 0.850 when adjusted for age and educational level (Figure 4C).

## 4. Discussion

In this cross-sectional study, we used a local 16-odor CSIT to assess olfactory identification ability in an aging Chinese population to assist in the early diagnosis of MCI and AD. Our results demonstrated that olfactory identification impairment occurred in 19.9% of the cognitively NCs, 52.7% of patients with MCI, and 69% of patients with AD. After adjusting for associated confounders, a lower CSIT score and higher olfactory impairment severity were identified as independent predictors of MCI or AD. The CSIT score was more effective in distinguishing patients with MCI and patients with AD from people with normal aging than in distinguishing patients with AD from patients with MCI. Our research also validated the use of the Chinese version of the CSIT in identifying cognitive decline among older Chinese adults with cognitive complaints. This test may serve as a new tool for the early detection of cognitive decline in clinical settings.

Olfactory function measurement involves multiple domains, including olfactory detection, intensity, familiarity, hedonics, discrimination, identification, and passive smelling (Good and Sullivan, 2015). Olfactory identification is recognized as one of the most commonly affected abilities in elderly people with cognitive impairment (Roalf et al., 2017). Olfactory identification deficit is most likely caused by damage to key brain structures that process olfactory information, such as the OB and the POC. These structures receive input from receptors in the nose and send signals to the OB and POC for further processing and identification of the odor (Devanand, 2016). A previous meta-analysis summarizing the structural changes implicated in olfactory processing showed that patients with AD and MCI had smaller OB volumes and a smaller POC when compared to controls and that these volumetric reductions could be measured as early as the MCI stage (Jobin et al., 2021a). Even altered olfactory identification function has been detected in subjective cognitive decline (SCD), a stage that occurs before MCI (Jobin et al., 2021b). A recent study provided evidence that the amygdala (a component of the POC) functions as a shared neural substrate linking olfactory identification ability with cognitive function (Shao et al., 2021; Tan et al., 2022). Olfactory identification deficits were also detected in both AD and MCI. A large-scale prospective cohort following 1,430 cognitively normal participants demonstrated that olfactory impairment was associated with the development of aMCI and progression to AD dementia (Roberts et al., 2016). Roalf et al. summarized published articles involving 1,993 patients with MCI and 2,861 healthy elderly adults and found that odor identification was one of the most impaired functions in MCI and was associated with the most prominent sensory deficit seen in AD (Roalf et al., 2017). Similarly, in our study, an olfactory identification deficit occurred in 52.7% of patients with MCI and 69% of patients with AD; these values were significantly higher than those in aging participants with normal cognitive function. Therefore, the olfactory deficit may represent a common manifestation accompanied by a decline in cognitive function.

As the world's population ages, cognitive impairment is becoming a significant global public health problem; early diagnosis and intervention are considered to be the most cost-effective measure to manage dementia, particularly in individuals with MCI. However, there is still a widespread misconception that cognitive impairment occurs naturally with age. According to the 2018 World Alzheimer's Disease Report, Alzheimer's disease is no longer a “senile disease,” and the age of onset has dropped from 65 to 55 years (Patterson, 2018). In the World Alzheimer's Report of 2021, global healthcare systems recommended providing annual screening of brain health for people over the age of 50 years (Gauthier et al., 2021). Moreover, it has been demonstrated that olfactory dysfunction is an initial symptom appearing years before cognitive decline and may serve as a clinical marker of AD's early stages and disease progression (Marin et al., 2018). Therefore, our study enrolled participants older than 50 years rather than the traditional threshold of 60 or 65 years, so that we could diagnose dementia promptly and treat it at an earlier stage. Several studies proved that olfactory function is affected by age, sex, culture, smoking, depression, and comorbidity (Yahiaoui-Doktor et al., 2019; Touliou et al., 2021). It has also been reported that there is a general decline in odor identification ability with aging, which accelerates significantly in individuals above 70 years of age (Devanand, 2016). Our study identified age and education as two confounders for the outcome. To identify the independent association between olfactory identification ability and cognitive function, we built two regression models by applying a stepwise adjustment of age, gender, and education; this was followed by the adjustment of other comorbidities. The analysis revealed the robust predictive role of the olfactory identification score for patients with MCI and AD.

Furthermore, we also observed that, in the subgroup analysis, aging did not affect olfactory function in normal controls; however, the CSIT score showed a stepwise reduction with aging in patients with MCI and AD, thus indicating an accelerated decline in olfactory function in these patients. Moreover, interaction effect analysis did not identify any significant interaction between educational level and olfactory effects, thus suggesting that the educational level did not alter the association between olfactory identification ability and cognitive function. Therefore, CSIT could be used as a predictor for the early detection of MCI or AD. In our study, CIST scores were significantly associated with MoCA and MMSE scores in the overall population and in the MCI and AD groups. This was particularly the case for MoCA scores, thus indicating that participants with good performance in cognitive function were more likely to achieve a higher CIST. These findings were consistent with those from a study by Lehrner who showed that the correlation coefficient between odor identification and MMSE score was 0.33 (Lehrner et al., 2009). In another study, Quarmley et al. reported that the MoCA score and odor identification performance were correlated in aMCI with a correlation coefficient of 0.24 (Quarmley et al., 2017).

Due to differences in geographic areas, ethnic races, and measuring modalities, a local measurement instrument carrying familiar odors with optimal discrepancy should be developed and validated. A cross-cultural study focusing on the performance of Sniffin' Sticks in Chinese and German patients with PD revealed that the adapted Chinese version of the test showed better resolution for PD (Pinkhardt et al., 2019). In the current study, we used a 16-odor local CSIT to detect the olfactory function of participants. A previous study demonstrated that this test has a test-retest reliability of 0.92 and has been validated against the UPSIT and the Sniffin' Sticks Identification Test 16 (SS-16) in a Chinese population, achieving a higher score (by 15% on average) than the other two tests (Feng et al., 2019). In our study, more than 90% of the adults with normal aging could correctly identify 12 of the 16 odors presented in this test, thus exhibiting a high identification rate for the included odors. Moreover, the test can be completed within 5 min and is widely accepted by aging adults due to its novelty, entertainment, and reliability. Therefore, a simple-to-administer odor identification test is recommended to screen individuals at risk of developing MCI or AD.

The accuracy of the CSIT for differentiating MCI and AD from people with normal aging was 0.738 and 0.812, respectively, with optimal thresholds of 13 and 12, respectively. However, we also noted that our olfactory test presented with a lower sensitivity and a higher specificity for distinguishing patients with MCI or AD from controls. Similarly, this issue was also evident in other related studies. Kim et al. reported that the modified Korean version of the Sniffin' Sticks test had a testing accuracy of 0.670 when distinguishing patients with MCI from NCs with a sensitivity of 0.462 at a cutoff point of 7 (Kim et al., 2020; Park et al., 2021). As described by Quarmley et al., Sniffin' Sticks were able to distinguish patients with MCI from normal individuals with an accuracy of 0.731, a sensitivity of 0.62, and a specificity of 0.73 at an optimal cutoff point; this test also distinguished AD from MCI with an accuracy of 0.67 (Quarmley et al., 2017). In another study, Wheeler et al. reported that the AUC of the ROC analysis for the San Diego Odor Identification Test when diagnosing patients with AD from controls was 0.63 (Wheeler and Murphy, 2021). Tahmasebi et al. reported that the sensitivity of impairment of olfactory identification for AD prediction was low at 46.2% but with a high specificity of 81.9% (Tahmasebi et al., 2019). Thus, these authors advocated that combining this tool with other neuropsychological tests might improve predictive accuracy. A systemic meta-analysis revealed that the MoCA scores yielded an AUC of 0.846 with a sensitivity of 80.48% and a specificity of 81.19% when predicting MCI with a cutoff point of 24/25. For MMSE, the more important cutoff was 27/28 (with a sensitivity of 66.34% and a specificity of 72.94% with an AUC of 0.736), with MoCA scores showing better screening performance than MMSE scores in MCI among patients over 60 years of age (Pinto et al., 2019; Jia et al., 2021). Therefore, the olfactory identification test may have comparable performance with the MMSE and may not be superior to MoCA scores when detecting MCI. However, this remains a promising tool for the clinical diagnosis of cognitive decline due to its indispensable characteristics. The application of MMSE and MoCA tests in clinical practice is always time consuming (>10 min) and requires a professional clinician or neuropsychologist to administer, which potentially results in additional medical costs and the presence of subjectivity and variability in diagnostic results (Yoo et al., 2020). Besides, their performance is affected by their educational years and the hearing or language abilities of the participants. Moreover, the MMSE scale exhibits low sensitivity in detecting earlier cognitive decline or prodromal dementia (MacAulay et al., 2017). CSIT is a non-invasive tool that can compensate, to some extent, for the shortages of the referred neuropsychiatric scales. The CIST device automatically scores within 10 min and does not require a deeply trained physician to operate, thus providing objective evidence to assist the clinical diagnosis. The CIST scores are scarcely affected by the level of education a person attains. Moreover, CSIT facilitates earlier detection of cognitive decline since olfactory dysfunction occurs several years before the onset of symptoms. Finally, the olfactory test is easy to use and implemented in primary care facilities, particularly in rural areas and communities, for early large-scale screening due to its novelty, reliability, high acceptance by participants, and low-cost characteristics. The olfactory measuring tools have disadvantages that need to be addressed. They are less sensitive than MoCA in detecting MCI and are not suitable for predicting cognitive impairment in patients having olfactory problems with other etiologies, except for aging and neurodegenerative diseases, such as head trauma, rhinitis, post-infectious sequelae from COVID-19, inhaled injuries, and the side effects of medications.

There are several limitations to this study that need to be considered. First, this study was limited due to its cross-sectional design. Thus, a dynamic long-term follow-up is recommended. Second, the involved participants were recruited at a single center in our hospital; thus, the conclusion should be extrapolated with caution. More representative participants in multiple centers and larger sample size are needed to validate the efficiency of CSIT. Third, we did not analyze the predictive value of CSIT for identifying the subtypes of MCI (aMCI or naMCI)/ as we did not perform a special memory test for assessing the performance and capacity of the memory domain, such as the Wechsler Memory Scale—Fourth Edition, the Clinical Memory Scale, or the Fuld Object Memory Test. Meanwhile, the MoCA and MMSE scales performed in this study are not sufficient to split MCI into naMCI or aMCI, although these scales contain memory domain assessment. The discriminative efficiency of CSIT for aMCI should be evaluated in further studies. Finally, careful consideration of genetic background, pathological changes, or clinical covariates will enhance our understanding of the mechanism of olfactory dysfunction during the progression of AD.

Our study demonstrated that patients with MCI and patients with AD exhibited a higher incidence of olfactory identification deficits and lower CSIT scores than individuals with normal aging. A lower CSIT score and higher olfactory dysfunction severity independently predicted the risk of MCI or AD even after adjusting for a wide array of confounders, such as age, gender, education, and comorbidity. Approximately 75% of the odors provided in the CSIT had a correct identification rate of over 90% in normal elderly adults. Patients with MCI and AD showed an accelerated decline in olfactory function. Our study found that the CSIT test is effective in distinguishing between patients with MCI and patients with AD from the general aging population. We recommend the use of the CSIT test in diagnosing AD in China due to its high acceptance, adaptability, novelty, and reliability.

## Data availability statement

The raw data supporting the conclusions of this article will be made available by the authors, without undue reservation.

## Ethics statement

The studies involving human participants were reviewed and approved by Affiliated Hospital of Northwest University (Xi'an No.3 Hospital). The patients/participants provided their written informed consent to participate in this study.

## Author contributions

YT, GZ, and WS conceived and revised the manuscript. XM and YM wrote the manuscript. YM, SD, CD, XL, and HT performed the experiment and data collection. XM, JZ, and QZ participated in the statistical analysis of the data. All authors read and approved the final version of the manuscript.
